# Large-Scale Surfactant-Free Synthesis of p-Type SnTe Nanoparticles for Thermoelectric Applications

**DOI:** 10.3390/ma10030233

**Published:** 2017-02-26

**Authors:** Guang Han, Ruizhi Zhang, Srinivas R. Popuri, Heather F. Greer, Michael J. Reece, Jan-Willem G. Bos, Wuzong Zhou, Andrew R. Knox, Duncan H. Gregory

**Affiliations:** 1 WestCHEM, School of Chemistry, University of Glasgow, Glasgow G12 8QQ, UK; Guang.Han@glasgow.ac.uk; 2 School of Engineering & Materials Science, Queen Mary University of London, London E1 4NS, UK; ruizhi.zhang@qmul.ac.uk (R.Z.); m.j.reece@qmul.ac.uk (M.J.R.); 3 Institute of Chemical Sciences and Centre for Advanced Energy Storage and Recovery, School of Engineering and Physical Sciences, Heriot-Watt University, Edinburgh EH14 4AS, UK; S.R.Popuri@hw.ac.uk (S.R.P.); J.W.G.Bos@hw.ac.uk (J.-W.G.B.); 4 EaStCHEM, School of Chemistry, University of St. Andrews, St. Andrews, Fife KY16 9ST, UK; hfg@st-andrews.ac.uk (H.F.G.); wzhou@st-andrews.ac.uk (W.Z.); 5 School of Engineering, University of Glasgow, Glasgow G12 8QQ, UK; Andrew.Knox@glasgow.ac.uk

**Keywords:** tin telluride, synthesis, structure, thermoelectrics, nanomaterials

## Abstract

A facile one-pot aqueous solution method has been developed for the fast and straightforward synthesis of SnTe nanoparticles in more than ten gram quantities per batch. The synthesis involves boiling an alkaline Na_2_SnO_2_ solution and a NaHTe solution for short time scales, in which the NaOH concentration and reaction duration play vital roles in controlling the phase purity and particle size, respectively. Spark plasma sintering of the SnTe nanoparticles produces nanostructured compacts that have a comparable thermoelectric performance to bulk counterparts synthesised by more time- and energy-intensive methods. This approach, combining an energy-efficient, surfactant-free solution synthesis with spark plasma sintering, provides a simple, rapid, and inexpensive route to p-type SnTe nanostructured materials.

## 1. Introduction

Sustainable energy generation and conversion technologies have received extensive, world-wide attention, because of ever-growing global energy demands, decreasing fossil fuel reserves, and the negative influence arising from the combustion of these fuels on the environment [1,2]. Thermoelectrics are capable of directly converting thermal energy into electric energy and therefore, can be applied to the generation of useful electricity from both waste heat and solar heat, in various applications. The thermoelectric conversion efficiency of a material is governed by its dimensionless figure of merit, *ZT* = *S^2^*σ*T/*κ, with *S*, σ, *T,* and κ representing the Seebeck coefficient, electrical conductivity, absolute temperature, and thermal conductivity, respectively [1,2,3,4,5,6,7]. Recently, tin chalcogenides have received extensive attention, due to their lead-free, less toxic nature [8,9,10,11,12]. Interestingly, SnTe resembles its Pb counterpart in terms of its crystal structure and band structure, suggesting that it has the potential to be an efficient thermoelectric material [13,14,15]. However, the high concentration of Sn vacancies in “pristine” SnTe creates a very high carrier concentration. This leads to p-type semiconductivity and, in fact, poor thermoelectric performance, with a low Seebeck coefficient and a high electronic component of the total thermal conductivity [16,17]. Recently, several approaches have been employed in order to significantly improve the thermoelectric performance of SnTe. For example, strategies such as band structure engineering, via the introduction of resonant impurity levels through In doping [18] and band convergence through Cd [19], Mg [20], Hg [21], Mn [22], or Ag alloying [23], have been applied to improve the power factor (*S^2^*σ) of SnTe. Equally, solid solution alloying [24,25], nanostructuring [18,19,25,26,27,28,29,30], introducing interstitial atoms [31], and all-scale hierarchical architecturing [22], have been used to minimise the thermal conductivity by enhanced phonon scattering, pushing *ZT* to ~1.1–1.3 at ~823–873 K [18,19,20,29], and ~1.3–1.4 at ~900–923 K [21,22,26]. Nonetheless, the synthesis of SnTe requires time-consuming and energy-intensive processes, such as the heating, melting and annealing of precursor materials at high temperatures (~973–1423 K) [19,20,21,22,23,24,25,26,28,29,30,31,32,33]. Ahead of any potential utilisation of SnTe in thermoelectrics, it is critical to develop an inexpensive and scalable synthesis approach that would allow SnTe to be produced with control over the structure and performance.

Monoliths which are consolidated directly from metal chalcogenide nanomaterials have been demonstrated to be efficient thermoelectrics, mainly due to the enhanced phonon scattering by nanostructures [34,35,36,37]. In contrast, chemical vapour deposition (CVD) can produce one- and two-dimensional nanostructures of metal chalcogenides, such as SnTe [38,39,40,41], which can facilitate nanodevice fabrication. Conversely, however, it is problematic to then scale up this synthesis to produce thermoelectrics in large quantities. Bottom-up solution methods enable the energy-efficient synthesis of metal chalcogenide nanostructures to be realised, while simultaneously controlling their crystal structure, particle dimensions, and morphology [34,35,36,37,42,43]. Organic surfactants are commonly used in solution synthesis, to control the morphology of nanostructures through surface modification. Such surfactants are adsorbed on the surfaces of the synthesised nanostructures and influence their electrical conductivity [42,44]. In contrast, surfactant-free solution synthesis can produce chalcogenide nanomaterials with cleaner surfaces, ensuring a higher electrical conductivity [45,46]. To date, the reported solution approaches for SnTe normally use surfactants and only produce small amounts of samples (<500 mg) [47,48,49,50,51]. Further, there are limited reports of the thermoelectric performance of SnTe nanomaterials synthesised by solution methods [52,53]. Given the potential to improve the properties of the telluride, it is important to develop new solution approaches to SnTe nanostructures and to investigate the thermoelectric performance of the pellets consolidated from these nanomaterials.

Herein, we demonstrate a one-pot surfactant-free aqueous solution approach for the fast and straightforward preparation of >10 g of SnTe nanoparticles, with short reaction durations. The phase-pure, surfactant-free nanoparticles can be sintered into dense compacts with a stable p-type conducting behaviour and relatively low thermal conductivity.

## 2. Results

### 2.1. Materials Synthesis

In a typical synthesis of SnTe nanoparticles, 100 mmol NaOH (Sigma-Aldrich Ltd., Dorset, UK, 99.99%) and 10 mmol SnCl_2_·2H_2_O (Sigma-Aldrich Ltd., Dorset, UK, 99.99%) were added to 50 mL deionised water (DIW) within a two-neck round-bottom flask, to yield a transparent Na_2_SnO_2_ solution. In parallel, 10 mmol Te (Sigma-Aldrich Ltd., Dorset, UK, 99.8%) and 20 mmol NaBH_4_ (AlfaAesar, Haverhill, MA, USA, 98%) were added to 50 mL DIW within a single-neck round-bottom flask, to prepare a purple 0.2 mol·L^−1^ NaHTe solution:

2Te + 4NaBH_4_ + 7H_2_O → 2NaHTe + Na_2_B_4_O_7_ + 14H_2_↑. (1)

**Scheme 1:**
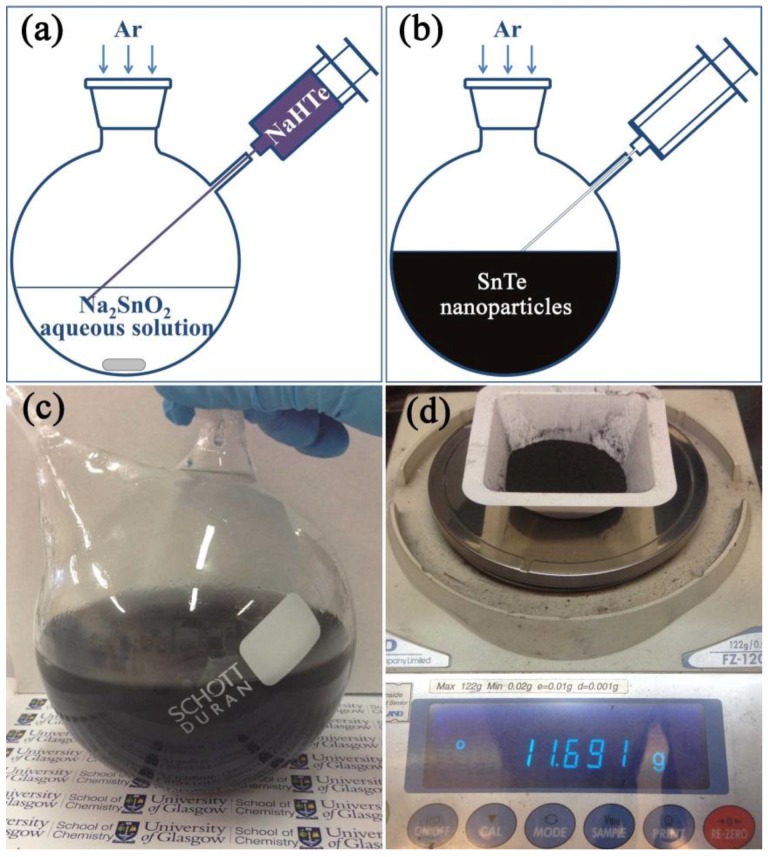
Solution synthesis of SnTe nanoparticles: schematic drawings showing (**a**) injection of NaHTe_(aq)_ into Na_2_SnO_2(aq)_ to trigger the reaction (NaHTe + Na_2_SnO_2_ + H_2_O → SnTe + 3NaOH) and (**b**) formation of SnTe nanoparticles; digital photographs showing (**c**) the nanoparticle solution after a scale-up synthesis and (**d**) a corresponding typical yield of SnTe nanoparticles (~11.7 g, ca. 95%) produced in such a one-pot synthesis.

After the Na_2_SnO_2_ solution was heated to its boiling temperature using an oil bath, the freshly prepared 50 mL NaHTe solution was promptly injected into the solution (1:1 Na_2_SnO_2_:NaHTe molar ratio for all experiments; Scheme 1a), leading to the immediate formation of a black precipitate (Scheme 1b). The mixture was heated to the boiling point, held for 2 h and allowed to cool to room temperature under Ar (BOC, 99.998%) on a Schlenk line. The products were collected by centrifuge, washed with DIW and ethanol several times and dried in an ambient atmosphere at 50 °C for 12 h. The amount of NaOH used (namely 0, 50, 100, and 150 mmol, corresponding to NaOH:SnCl_2_ molar ratios of 0, 5, 10, and 15, respectively) and the duration of the boiling process (5 min, 2 h, 24 h), were independently changed to investigate their effects on the synthesis. Scaled-up syntheses were performed with five-fold precursor amounts, i.e., using 500 mmol NaOH, 50 mmol SnCl_2_·2H_2_O, 50 mmol Te, and 100 mmol NaBH_4_ (Scheme 1c); the products, with a typical yield of ~11.7 g (Scheme 1d), demonstrated phase purity and a morphology identical to the products synthesised on a lower scale. The synthesised samples used for the characterisation and performance evaluation were stored in an Ar-filled MBraun glove box (<0.5 ppm H_2_O, <0.5 ppm O_2_), to avoid possible reactions with ambient air.

### 2.2. Materials Characterisation

The phase composition and crystal structures of the as-prepared samples were investigated by powder X-ray diffraction (PXD), using a PANalytical X’pert Pro MPD diffractometer in Bragg-Brentano geometry (Cu Kα_1_ radiation, λ = 1.5406 Å). Diffraction data were collected at room temperature with a step size of 0.017 ° over 10 ° ≤ 2θ ≤ 90 ° for 1 h. The crystal structures of the synthesised products were refined via the Rietveld method against PXD data, using the GSAS and EXPGUI software packages [54,55], with the previously published SnTe structure as a basis [56]. For the refinement, a shifted Chebyschev function (type 1 within GSAS) and a Pseudo-Voigt profile function (type 2 within GSAS) were applied to model the background and peak shape, respectively.

The morphological and chemical characteristics of the synthesised products were investigated by scanning electron microscopy (SEM, Carl Zeiss Sigma, 5 and 20 kV for imaging and elemental analyses, respectively), equipped with energy-dispersive X-ray spectroscopy (EDS, Oxford Instruments X-Max 80, Oxford Instruments, Oxfordshire, UK). The synthesised powders were spread onto a conductive carbon tape that was mounted on a standard SEM sample stub. The microstructure and crystal structure were further characterised by transmission electron microscopy (TEM) using a JEOL JEM-2011 electron microscope fitted with a LaB_6_ filament, operating at an accelerating voltage of 200 kV. TEM images, high resolution TEM (HRTEM) images and selected area electron diffraction (SAED) patterns were recorded using a Gatan 794 Multiscan CCD camera. The SnTe powders were dispersed in ethanol by sonication for 30 s, to obtain a uniform dispersion. Two to five drops of the suspension were dropped onto a 3 mm diameter holey C-coated Cu TEM grid. Thermogravimetric-differential thermal analysis (TG-DTA) of the samples was performed using a Netzsch STA 409 thermal analyser located in an Ar-filled MBraun glove box (<0.1 ppm H_2_O, <0.1 ppm O_2_). Approximately 25 mg of SnTe pellet was heated to 700 °C in an alumina pan under flowing Ar (60 mL·min^−1^), at a heating rate of 5 °C·min^−1^.

### 2.3. Materials Performance Evaluation

To measure the thermoelectric performance of SnTe nanoparticles, SnTe powders were loaded into a graphite die (diameter of 15 mm) within an Ar-filled glovebox, and then sintered using a spark plasma sintering (SPS) furnace (FCT HP D 25, FCT System GmbH) into dense pellets (relative density of >98%) at 450 °C for 5 min, in a vacuum (~4 Pa) with a uniaxial pressure of 50 MPa. The Seebeck coefficient (*S*) and electrical conductivity (σ) of the pellets were measured simultaneously, using a Linseis LSR-3 instrument under a helium atmosphere, within a temperature range of 300–530 K. The uncertainty in the measurement of *S* and σ are ~5%, leading to ~10% uncertainty for the thermoelectric power factor (*S^2^*σ) measurement. The thermal conductivity (κ) of the pellets was calculated using κ = *DC_p_*ρ, where *D*, *C_p_,* and ρ are the thermal diffusivity coefficient, specific heat capacity, and density, respectively. *D* was measured using a Linseis LFA 1000 instrument in a vacuum, within a temperature range of 300–530 K. *C_p_* values for pellets were calculated from the weighted average of the reported values of *C_p_* for SnTe [18] and SnO_2_ [57] and the Dulong-Petit limit of Te [58] (where the respective weights were determined from the refined phase fractions from the Rietveld refinements against PXD data). The density, ρ, of the pellets was measured by the Archimedes method. The uncertainty in the κ measurement is ~8%. The electronic thermal conductivity (κ*_e_*) was estimated by the Wiedemann-Franz law (κ*_e_* = *L*σ*T*, where *L* was estimated according to *L = exp (−S/116)* [59]; *S* values are positive in this study) and the lattice thermal conductivity (κ*_L_*) was calculated by subtracting κ*_e_* from κ. *ZT* was calculated using *ZT* = *S^2^*σ*T/*κ, with an uncertainty of ~15%.

## 3. Results and Discussion

**Figure 1:**
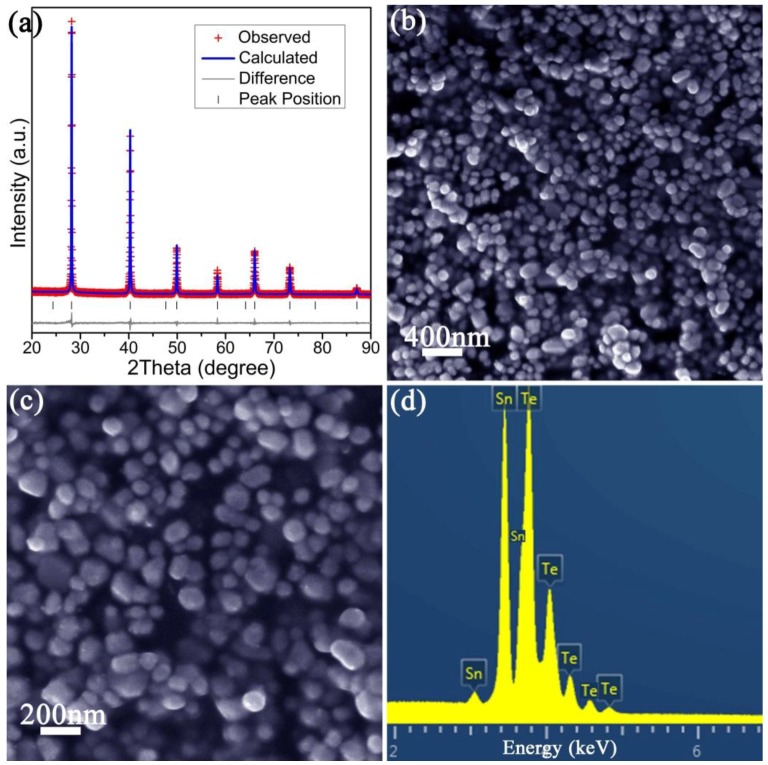
Characterisation of SnTe nanoparticles synthesised after 2 h: (**a**) Profile plot from Rietveld refinement against PXD data; (**b**,**c**) SEM images; (**d**) EDS spectrum.

The injection of a NaHTe solution into aqueous Na_2_SnO_2_ leads to the immediate formation of a precipitate of SnTe (Scheme 1a,b). Boiling the suspension for 2 h generates ˃10 g crystalline, phase-pure SnTe nanoparticles (Scheme 1c,d). The PXD pattern (Figure 1a) shows that all of the reflections can be exclusively indexed to cubic SnTe (ICDD card No. 65-0319) [60]. Rietveld refinement against the PXD data (Figure 1a; Table 1 and Table 2) confirms that the SnTe product crystallises in the cubic space group $Fm\bar{3}m$, with *a* = 6.3234(1) Å. SEM images (Figure 1b,c) reveal that the product exists as approximately isotropic nanoparticles, with individual diameters of 60–160 nm and an average diameter of ~95 nm. EDS spectra (Figure 1d) taken across the samples as point and area scans, consistently generate Sn:Te atomic ratios of 51.5(5):48.5(5).

**Figure 2:**
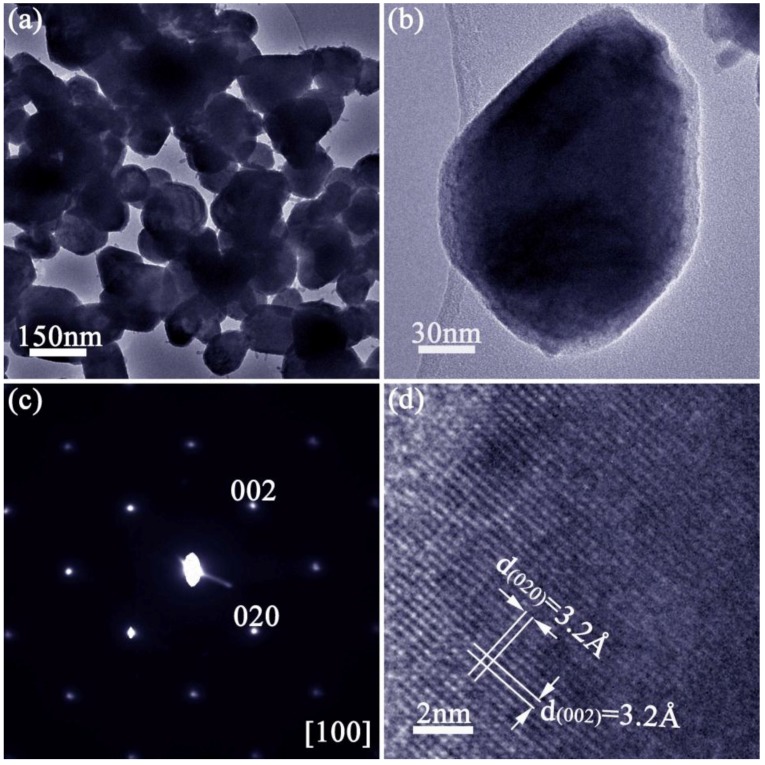
TEM characterisation of SnTe nanoparticles synthesised after 2 h: (**a**,**b**) TEM images of a group of nanoparticles and a single particle respectively; (**c**) SAED pattern collected from the particle in (**b**); (**d**) corresponding HRTEM image taken from the same particle.

TEM images (Figure 2a,b) confirm that the SnTe nanoparticles are 60–160 nm across. An exemplar SAED pattern (Figure 2c) collected from an isolated nanoparticle reveals the single crystalline nature of the particle and can be indexed along the [100] zone axis. A set of lattice spacings of 3.2 Å, intersecting at 90°, can be measured from the corresponding HRTEM image (Figure 2d), and correspond to the {002} lattice spacings, confirming the high crystallinity of the SnTe nanoparticles.

**Figure 3:**
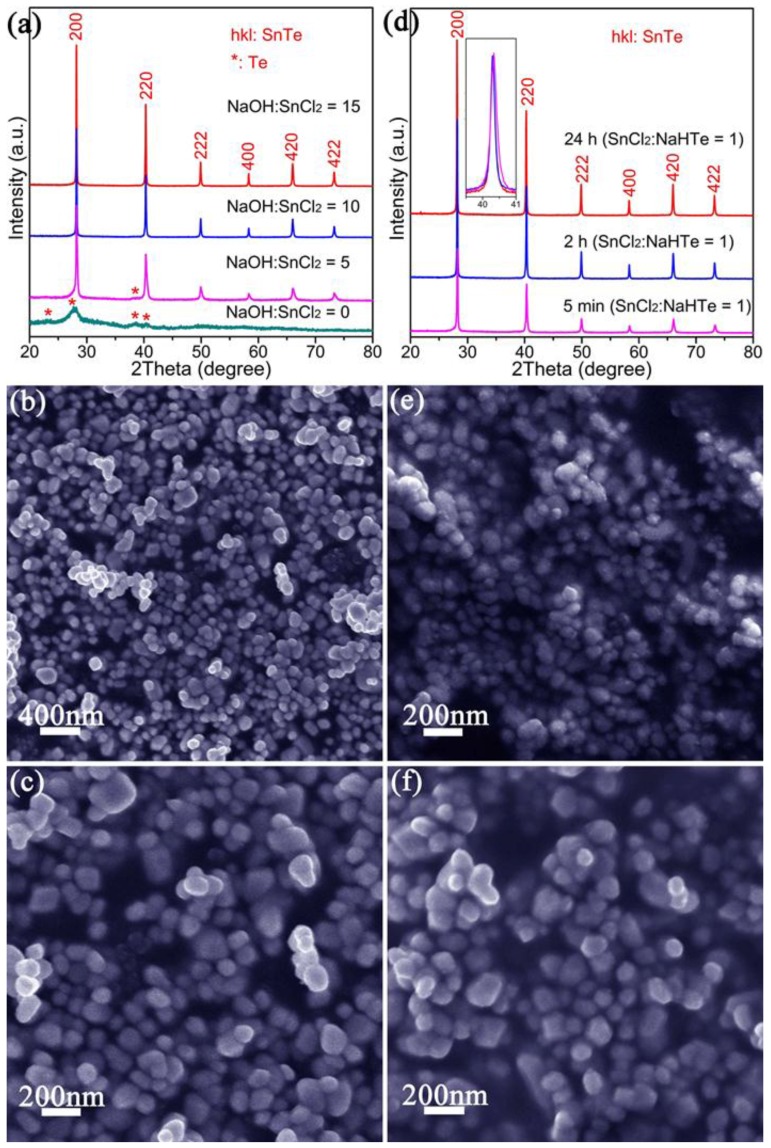
Characterisation of the products synthesised under varied conditions: (**a**) PXD patterns of products synthesised over 2 h with different NaOH:SnCl_2_ precursor molar ratios (namely 0:1, 5:1, 10:1, 15:1); (**b**,**c**) SEM images of the SnTe nanoparticles synthesised over 2 h with a precursor molar ratio of 15:1; (**d**) PXD patterns of 10:1 NaOH:SnCl_2_ products synthesised over different reaction durations (namely 5 min, 2 h, 24 h) (the inset shows the (220) peaks in a magnified section of the diffraction pattern); (**e**,**f**) SEM images of the 10:1 NaOH:SnCl_2_ nanoparticles synthesised in 5 min and 24 h, respectively.

A controlled synthesis of phase-pure SnTe depends on certain, well-defined experimental parameters. For example, the NaOH concentration is a critical synthesis parameter for the synthesis of pure, crystalline SnTe nanoparticles. Without the addition of NaOH, the obtained product is poorly crystalline and contains a large fraction of Te impurity (Figure 3a). By contrast, when increasing the NaOH:SnCl_2_ molar ratio to 5:1, the phase fraction of Te significantly decreases, accompanied by a sharp increase in the amount of SnTe (Figure 3a). At higher ratios (10:1, 15:1), phase-pure, highly crystalline SnTe can be obtained (Figure 3a). Clearly, increasing the NaOH concentration leads to SnTe with a higher purity. NaHTe can be easily oxidised into Te. Therefore, it is reasonable to deduce that NaOH can promote the disproportionation of Te into Te^2−^ and TeO_3_^2−^, to eliminate this Te impurity and to drive the formation of SnTe to completion [47,52], probably via:

3Te + 6NaOH → 2Na_2_Te + Na_2_TeO_3_ + 3H_2_O. (2)

SEM images (Figure 3b,c) reveal that the product synthesised with a NaOH:SnCl_2_ ratio of 15:1, forms as nanoparticles with a mean size of ~115 nm across, which is slightly larger than the particle size obtained with a hydroxide:chloride ratio of 10:1 (Figure 1). As might be expected, the duration of the reaction provides another means to tune the SnTe particle size. Phase-pure SnTe persists over 5 min and 24 h reaction durations (Figure 3d), but the average particle sizes of the telluride can be tuned from ~65 to ~115 nm across, respectively (Figure 3e,f).

**Figure 4:**
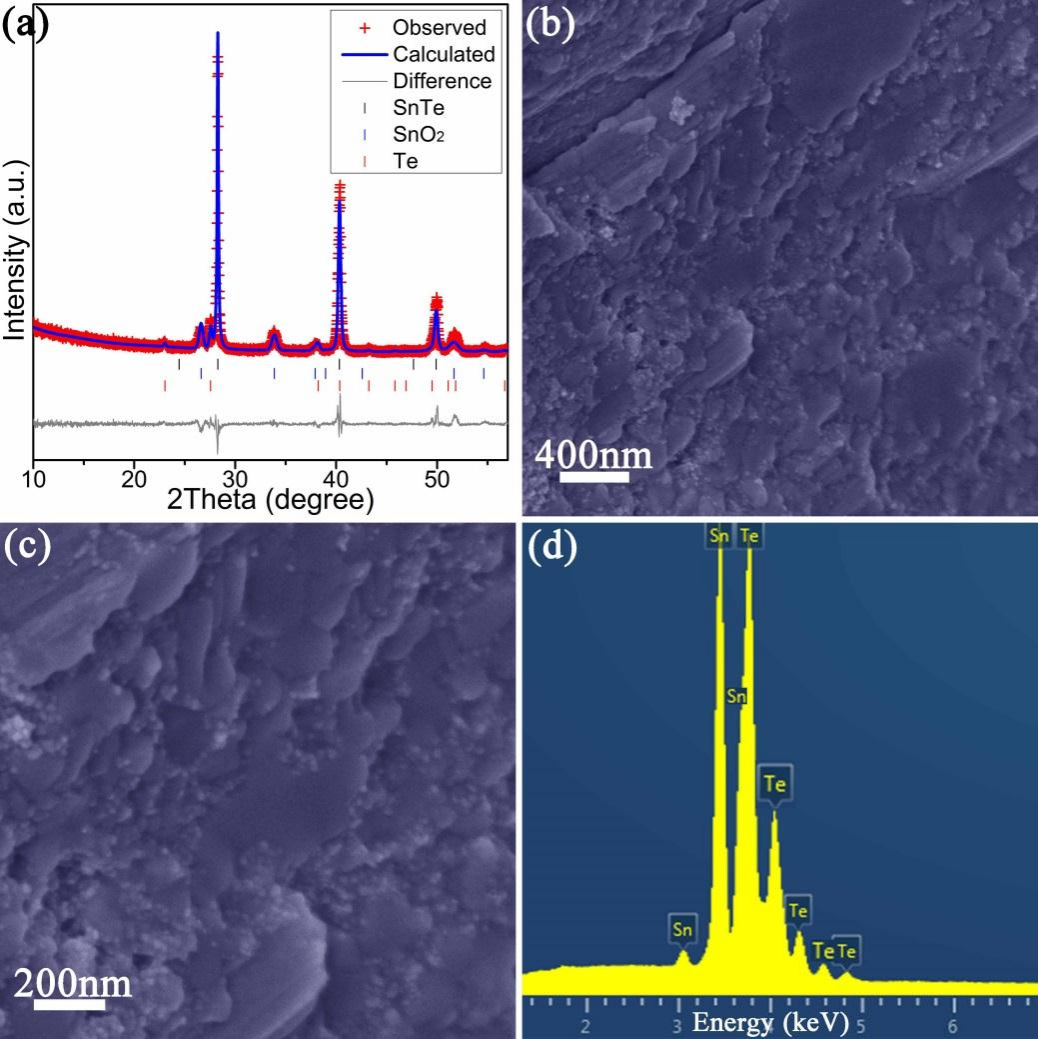
Characterisation of the SnTe pellet: (**a**) Profile plot from Rietveld refinement against PXD data; (**b**,**c**) SEM images; (**d**) EDS spectrum.

The ability to prepare SnTe nanostructures in >10 g quantities repeatedly allowed the facile fabrication of SnTe dense pellets, via SPS techniques. Pellets with ~98% of the theoretical SnTe density, sintered from 2 h solution-synthesised nanomaterials (10:1 NaOH:SnCl_2_), were obtained. Rietveld refinement against the PXD data (Figure 4a, Table 3) shows that the pellets consist of a main phase of cubic SnTe and two minority phases of tetragonal SnO_2_ and trigonal Te (72.3(1) wt % SnTe − 20.4(3) wt % SnO_2_ − 7.3(2) wt % Te). This indicates that SnTe nanoparticles were partially oxidised and decomposed into SnO_2_ and Te during the SPS process at a high sintering temperature, probably through the following reaction:

SnTe + O_2_ → SnO_2_ + Te. (3)

**Figure 5:**
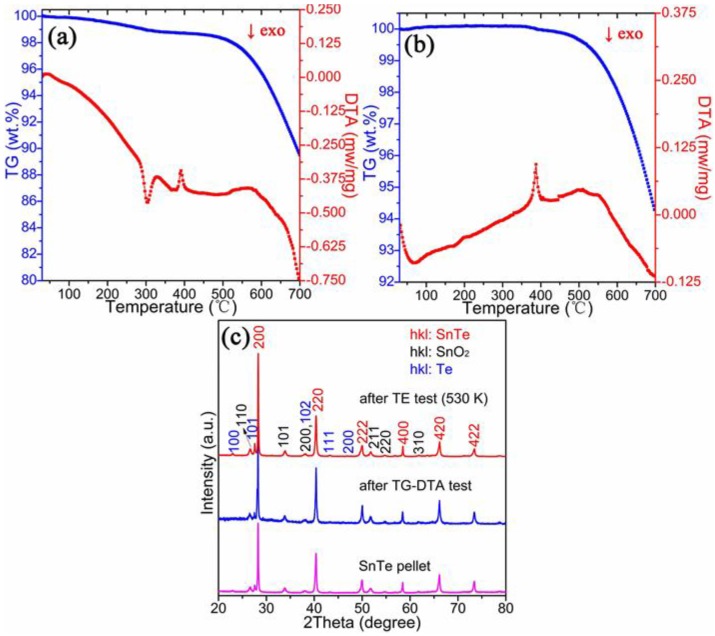
(**a**,**b**) TG-DTA profiles of the SnTe powder and pellet, respectively; (**c**) PXD patterns of an SnTe pellet before and after TG-DTA (to 973 K) and thermoelectric property testing (to 530 K).

This exothermic oxidation process and an ensuing endothermic Te melting process have been detected at ~570 K and ~660 K, by thermogravimetric-differential thermal analysis (TG-DTA) of SnTe powder under an Ar flow (Figure 5a). The melting point of Te in this study is lower than that typically reported for bulk Te (~723 K), which is likely to be due to the small size of the Te product particles generated by the oxidation (Equation (3)) [61]. A similar oxidation/decomposition process has been observed in SnSe pellets which have been hot pressed from surfactant-free SnSe nanoparticles [43]. There is an expectation that the reactive surfaces of the small nanoparticles adsorb O_2_, contributing to the oxidation to SnO_2_ [43]. SEM images (Figure 4b,c) reveal that the pellets are constructed from a “matrix” of densely packed nanoparticles, with smaller (~20 nm) nanoparticles almost uniformly distributed within this “matrix”. Similar nanoparticles with sizes of ~5–20 nm were formed when surfactant-free SnSe nanoparticles were hot pressed into dense pellets and these ~5–20 nm particles were identified as SnO_2_ [43]. Considering that the as-synthesised SnTe nanoparticles are 60–160 nm across, we deduce that the smaller nanoparticles distributed across the internal structure of the sintered SnTe pellet most likely belong to SnO_2_ and/or Te. EDS otherwise confirms that the Sn:Te atomic ratio is ~55.5(5):45:5(5) (Figure 4d) in the pellet. Based on the phase fractions from the Rietveld refinement against the PXD data and the elemental analyses from EDS, it is estimated that about ~32% of the original SnTe nanoparticles decomposed/oxidised, with ~58% of the Te produced in this reaction (Equation (3)) vaporising during SPS. TG-DTA of an SnTe pellet under an Ar flow shows a negligible weight loss below 620 K, but suggests that thermal decomposition is initiated above this temperature (Figure 5b). As for the pellet sample, an endothermic peak was observed at ~660 K, which could be related to the melting of Te impurity in the pellet. In contrast to the TG-DTA of the powder sample, however, TG-DTA of the SnTe pellet presents no evidence of an exothermic peak at ~570 K, indicating no further oxidation and decomposition during heating of the SnTe pellet. PXD of the pellet after TG-DTA (Figure 5c) shows the presence of the same phases prior to thermal analysis, with very similar phase fractions. The maximum temperature for the thermoelectric measurements in this work was restricted to 530 K. By taking thermal and electrical data from 300 to 530 K, we could therefore guarantee that the measurement region represented one of stability for the material. Indeed, PXD data for the pellet demonstrated that the phase compositions and fractions were unchanged after thermoelectric evaluation (Figure 5c).

**Figure 6:**
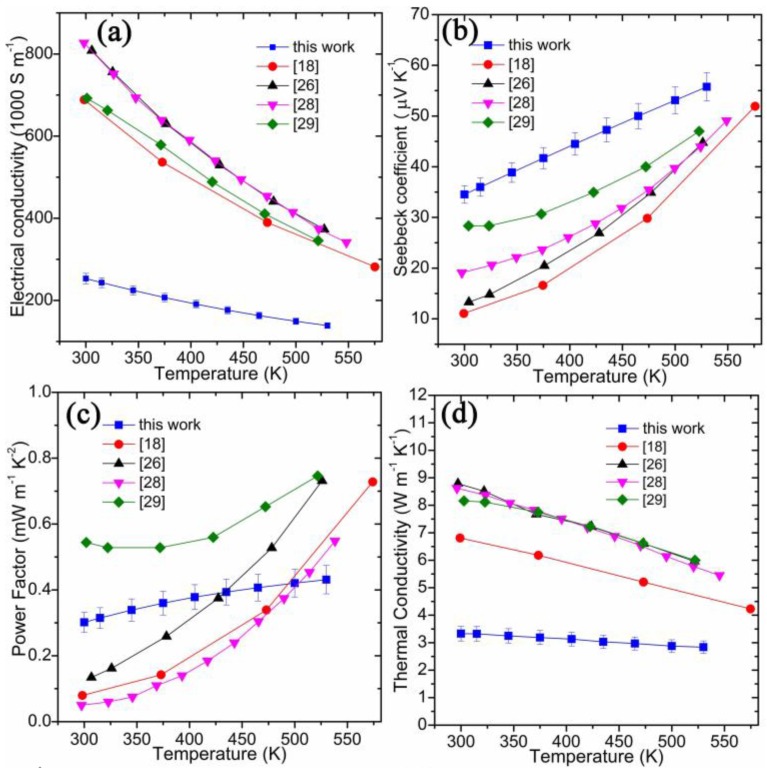

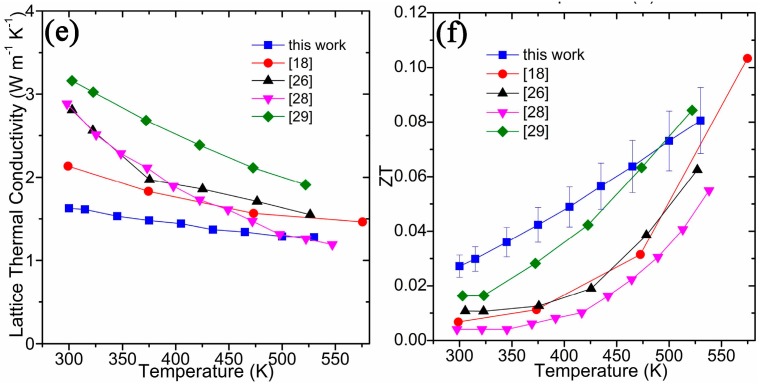
Thermoelectric properties of a SnTe pellet showing: (**a**) the electrical conductivity; (**b**) the Seebeck coefficient; (**c**) the power factor; (**d**) the thermal conductivity; (**e**) the lattice thermal conductivity and (**f**) *ZT* as a function of temperature, with comparisons with selected literature values for SnTe bulk materials synthesised by ball milling and high-temperature synthesis.

The electrical conductivity (σ) of the pellet decreases from ~2.53 × 10^5^ S·m^−1^ at 300 K, to ~1.39 × 10^5^ S·m^−1^ at 530 K (Figure 6a), demonstrating the behaviour typical of a degenerate semiconductor, that could be related to a low but significant concentration of intrinsic Sn vacancies in the SnTe structure [16,17]. *S* increases from ~34.5 μV·K^−1^ at 300 K, to ~55.8 μV·K^−1^ at 530 K (Figure 6b). It should be noted that σ and *S* are relatively low and high, respectively, compared to those of SnTe bulk materials synthesised by high energy ball milling [18] and high-temperature methods [26,28,29], which could result from the influence of impurities (i.e., SnO_2_ and Te) and/or the relatively low Sn vacancy concentration (no deviation in the Sn site occupancy from unity could be identified by Rietveld refinement, for example, which would support this premise). The power factor (*S^2^*σ) of SnTe increases from ~0.30 mW·m^−1^·K^−2^ at 300 K, to ~0.43 mW·m^−1^·K^−2^ at 530 K (Figure 6c). These figures are comparable to those for SnTe synthesised by high energy ball milling [18] and via high-temperature methods [26,28,29] (Figure 6c). The SnTe pellets display relatively low thermal conductivity (κ), and the values decrease from ~3.33 W·m^−1^·K^−1^ at 300 K, to ~2.84 W·m^−1^·K^−1^ at 530 K (Figure 6d). The corresponding lattice thermal conductivity (κ*_L_*) decreases from ~1.63 W·m^−1^·K^−1^ at 300 K, to ~1.28 W·m^−1^·K^−1^ at 530 K (Figure 6e), which is lower when compared to bulk materials [18,26,28,29], and could be due to the nanostructuring of the SnTe pellets. *ZT* was derived from the electrical and thermal property results and increases from ~0.027 at 300 K, to ~0.081 at 530 K (Figure 6f). These *ZT* values are comparable to those obtained previously from SnTe bulk materials prepared via high temperature synthesis (e.g. heating, melting, and annealing precursors at ~973–1423 K for long durations) [26,28,29], or by high energy ball milling [18]. This demonstrates that our surfactant-free solution approach is an energy-efficient and large-scale method for synthesising SnTe, with a comparably high thermoelectric performance. Previous research has demonstrated that the power factor in SnTe can be significantly enhanced through band structure engineering, such as introducing resonant impurity levels through In doping [18] and band convergence through Cd [19], Mg [20], Hg [21], Mn [22] or Ag alloying [23]. Therefore, by introducing these dopants into SnTe nanoparticles as part of our solution synthesis process, improved values for *S^2^*σ and *ZT* can be expected. Judicious doping and substitution is also likely to provide a means to stabilise the cubic SnTe phase to decomposition at elevated temperature. These are aspects that we are currently investigating.

## 4. Conclusions

In summary, we have developed a simple, quick, and inexpensive one-pot solution route for synthesising SnTe nanoparticles in gram quantities (>10 g per run for a 2 h growth). Dense, nanostructured SnTe pellets have been fabricated by spark plasma sintering with a thermoelectric performance comparable to SnTe bulk materials synthesised by energy-intensive methods. As a similar solution method has already been applied to synthesise high-performance SnSe nanomaterials [45] and can be extended to SnS (to be reported soon), such a surfactant-free approach has been demonstrated to be an efficient, versatile method for synthesising efficient Sn chalcogenide thermoelectric nanomaterials.

## Figures and Tables

**Table 1:** Crystallographic data for SnTe nanoparticles synthesised after 2 h boiling.

Chemical Formula	SnTe
Crystal System	Cubic
Space Group	$Fm\bar{3}m$
*a* (Å)	6.3234(1)
Volume (Å^3^)	252.84(1)
Z	4
Formula Weight (g·mol^−1^)	246.29
Calculated density (g·cm^−3^)	6.470
R_wp_	0.1181
R_p_	0.0832
χ^2^	1.449

**Table 2:** Atomic parameters for SnTe nanoparticles synthesised after 2 h boiling.

Atom	Wyckoff Symbol	x	y	z	100*U_iso_/Å^2^	Occupancy
Sn	4a	0	0	0	3.87(8)	1
Te	4b	0.5	0.5	0.5	2.49(7)	1

**Table 3:** Crystallographic data for SnTe pellet.

Chemical Formula	SnTe	SnO_2_	Te
Crystal System	Cubic	Tetragonal	Trigonal
Space Group	*Fm*$\bar{3}$*m (225)*	*P4_2_/mnm (136)*	*P3_1_21 (152)*
*a* (Å)	6.3441(7)	4.7610(10)	4.4836(16)
*b* (Å)			
*c* (Å)		3.2008(10)	5.9551(34)
Volume (Å^3^)	255.33(8)	72.55(3)	103.67(5)
Z	4	2	3
Formula Weight (g·mol^−1^)	246.29	150.69	127.6
Calculated density (g·cm^−3^)	6.407	6.898	6.131
Phase fraction (wt %)	72.3(1)	20.4(3)	7.3(2)
R_wp_	0.1497		
R_p_	0.1120		
χ^2^	2.359		
